# Hypogastric Artery Occlusion with Evoked Potentials Monitoring as Bailout Technique to Assess the Risk of Postoperative Spinal Cord Ischemia

**DOI:** 10.1055/s-0039-1687866

**Published:** 2019-07-22

**Authors:** Mario D'Oria, Cristiano Calvagna, Stefano Chiarandini, Barbara Ziani

**Affiliations:** 1 Vascular and Endovascular Surgery Unit, Cardiovascular Department, University Hospital of Cattinara ASUITS, Trieste (TS), Italy

**Keywords:** hypogastric artery, spinal cord ischemia, endovascular aortic surgery, acute aortic dissection, evoked potentials

## Abstract

A 65-year-old man, with previous open surgical repair of an infrarenal abdominal aortic, presented with acute complicated (paraplegia) Type B aortic dissection. He successfully underwent endovascular repair of the descending thoracic and abdominal aorta. Following the procedure, the neurological manifestations resolved. As he had a concomitant aneurysm of the right hypogastric artery (HGA), we executed a 10-minute balloon occlusion of this artery with evoked potential measurements to assess the risk of spinal cord ischemia after exclusion of the right HGA. The examination was interpreted as negative, and we proceeded with coil embolization of the right HGA and subsequent placement of an endograft landing distally within the external iliac artery. The postoperative course was totally uneventful, and the patient was discharged home 4 days after the operation. Computed tomography angiography follow-up at 1, 6, 12 and 24 months showed patency of all endografts without any signs of endoleak and effective remodeling of the descending thoracic aorta with volume reduction of the false lumen.

## Introduction

The advent of endovascular techniques has radically altered the treatment algorithm of most descending thoracic and thoracoabdominal aortic pathologies over the past 20 years. Endovascular repair, for both elective and emergent cases, has become particularly accepted as the first-line approach in high-risk patients who would not otherwise be able to tolerate open surgical repair without a significant risk of morbidity and/or mortality. 1 2

Nevertheless, the management of aortoiliac aneurysms, and the associated clinical significance of preserving or sacrificing the hypogastric artery (HGA), still continues to represent a matter of debate. 3 4

We report a case of staged endovascular treatment of a patient with complex aortoiliac pathology with preservation of only the left HGA. We determined the likelihood of postoperative spinal cord ischemia (PSCI) to be low by means of dynamic angiography with associated neuromonitoring. The patient has not developed any neurologic complication at 24 months' follow-up.

## Case Presentation

**Fig. 1:**
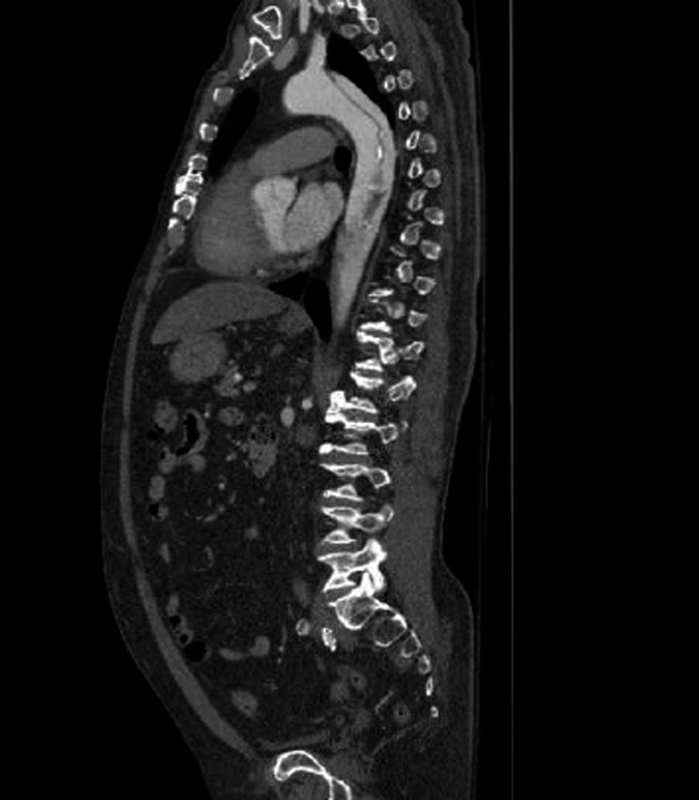
Computed tomography angiography showing the entry tear of the dissection just below the origin of the left subclavian artery.

**Fig. 2:**
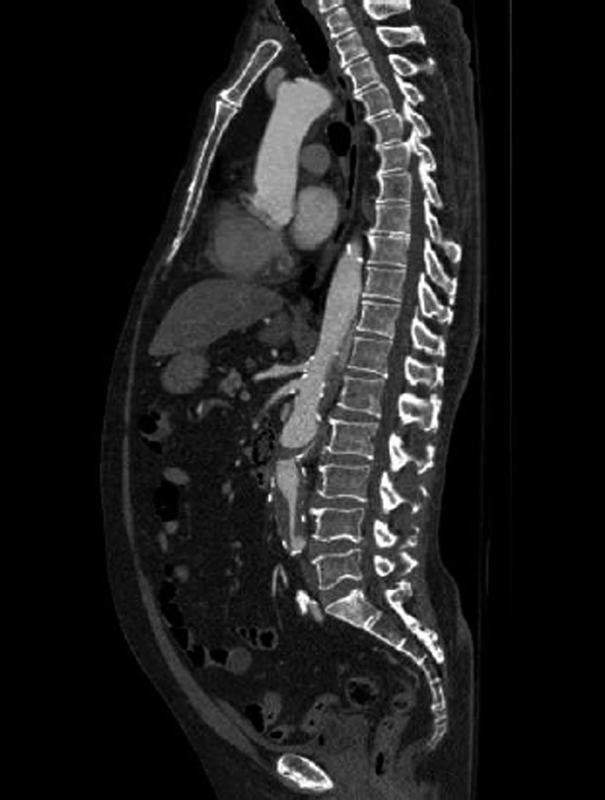
Computed tomography angiography showing the subtotal occlusion of the aortic lumen just below the level of the renal arteries.

A 65-year-old male presented to the emergency department after the acute onset of paraplegia associated with severe interscapular pain and hypertensive crisis. The patient had previously undergone open surgical repair of an infrarenal abdominal aortic aneurysm with aorto-aortic reconstruction sutured proximally approximately 15 mm below the lowest (right) renal artery and distally to the aortic bifurcation. Computed tomography angiography (CTA) showed the presence of an acute Stanford Type B aortic dissection originating just below the ostium of the left subclavian artery (LSA) and extending downward to the level of the renal arteries. Another radiological finding was a right HGA aneurysm measuring approximately 40 mm in maximal transverse diameter. The main aortic visceral branches (celiac trunk, superior mesenteric artery, renal arteries) were all patent and all took off from the true lumen. The abdominal aorta was almost completely occluded for a short segment just below the level of the renal arteries. This finding could be explained, in our opinion, by reflection and overturning of the dissection flap over the suture line of the preexisting surgical graft. The two iliac arteries were otherwise reperfused through the pelvic collateral network ( Figs. 1 and 2 ).

The patient was immediately transferred to the operating theater and underwent endovascular repair of the descending thoracic and abdominal aorta. After bilateral surgical cutdown of the common femoral arteries (CFAs), we gained access to the true lumen above the renal arteries using combined angiographic and transesophageal echocardiographic control. At first, we covered the proximal entry tear with placement of a thoracic endograft (Valiant, MEDTRONIC) without any oversizing. Proximal and distal landing zones were achieved respectively in Ishimaru's zone 2 of the aortic arch and approximately 10 cm above the ostium of the celiac trunk. We then proceeded with placement of a bifurcated abdominal endograft (Endurant II, MEDTRONIC) with 15% oversizing. Proximal landing zone was achieved just below the right renal artery while distal landing zone was reached, bilaterally, above the bifurcation of the common iliac artery (CIA). At the end of the procedure, no residual filling of the false lumen was noted. Owing to the patient's unstable clinical condition, we decided not to perform any adjunctive procedure (i.e., restoration of blood flow to the LSA or exclusion of the right CIA aneurysm) at this time and the patient was transferred to the intensive care unit.

Following the operation, the patient's condition improved and he fully recovered from paraplegia. He was discharged home 4 weeks after the index procedure. CTA performed at this time demonstrated correct placement of the endografts with adequate exclusion of the false lumen, patency of all aortic side branches, and absence of any detectable endoleak. At this time, the patient was also screened for the most common genetic aortopathies but the tests were negative.

**Fig. 3:**
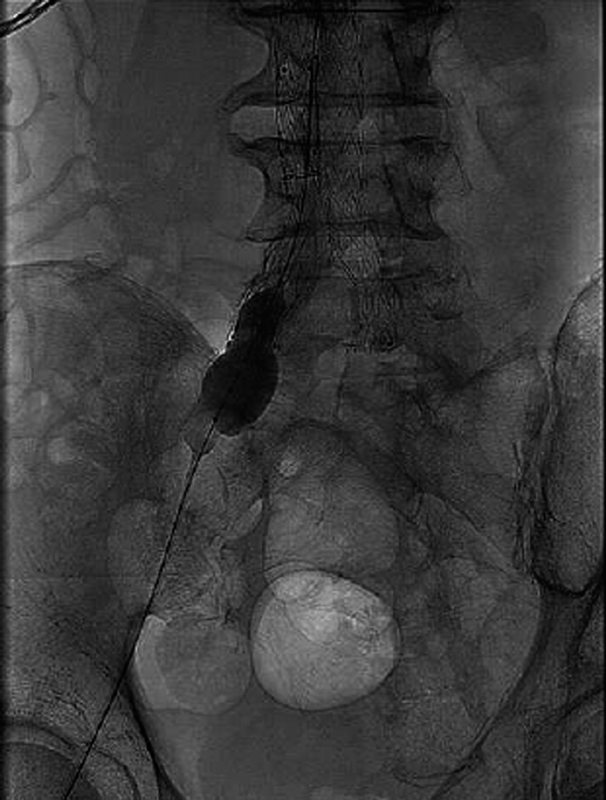
Balloon occlusion of the right hypogastric artery during evoked potentials monitoring.

We scheduled elective exclusion of the right HGA aneurysm 4 months after the index event. Open repair of the iliac aneurysm with HGA preservation was ruled out because of the patient's preference for endovascular treatment. As the patient's anatomy was unsuitable for HGA preservation, we planned for its exclusion. We assessed the likelihood of PSCI through dynamic angiography associated with neuromonitoring. In the operating room, the patient was positioned supine and general endotracheal anesthesia was administered. The right HGA was thereafter occluded for 10 minutes ( Fig. 3 ), with simultaneous recording of motor-evoked potentials (MEPs) and somatosensory-evoked potentials (SSEPs) bilaterally on the legs. Systemic heparinization was not administered during the balloon occlusion test. Our neurophysiologist interpreted the examination as negative for any signs of ischemia of the spinal cord, and thus we proceeded with coil embolization of the right HGA and subsequent positioning of a straight iliac endograft (Endurant II, MEDTRONIC) whose distal landing zone was achieved in the external iliac artery, covering the orifice of the HGA. The postoperative course was uneventful and the patient was discharged home 4 days after the operation.

CTA follow-up at 1, 6, 12 and 24 months showed satisfactory placement of all endografts without any signs of endoleak or endograft failure. There was effective remodeling of the descending thoracic aorta, with volume reduction of the false lumen. The patient was free from new-onset neurologic and/or aortic complications at the longest available clinical follow-up.

## Discussion

Sacrifice of the HGA for effective treatment of complex aortoiliac pathologies is not without sequelae. Several studies have shown that unilateral or bilateral HGA occlusion can be performed in most cases without consequent life-threatening pelvic ischemic complications, but a significant number of patients can develop symptoms, the most common being buttock/thigh claudication and new-onset erectile disfunction. 5 Among the postoperative complications of HGA sacrifice, the most feared is PSCI which can occur in up to 2 to 4% of patients. 6 7 Thus, despite previously mentioned studies supporting HGA occlusion as a relatively innocuous procedure, it is well documented that pelvic ischemic complications can actually occur. To date, there is no clear established preoperative strategy to assess the likelihood of PSCI to occur after bilateral or unilateral HGA exclusion.

Indeed, PSCI is the most feared and dramatic complication of descending thoracic and thoracoabdominal aortic procedures which has not been completely eliminated by endovascular repair. 8 The risk of PSCI increases with the extent of the aneurysm, length of the stent graft used, preexisting aortic reconstruction, and occlusion of LSA or HGA. 9 10 Other risk factors for the development of PSCI, which can represent relative indications for HGA preservation, are young age, left ventricular dysfunction, severe atherosclerotic disease of the superior mesenteric artery or the ipsilateral CFA or contralateral HGA, and presence of a large patent inferior mesenteric artery. 11 In a recent review, it was shown that higher rates of PSCI seem to be associated with extensive endovascular procedures, particularly with concomitant thoracic aneurysm repair, even when only a single collateral was occluded. 12 In that sense, preservation of HGA perfusion is important to minimize the risk of spinal cord injury because its patency significantly reduces the incidence of PSCI after extensive aortic endografting. 13 14

Attempts at preserving HGA patency should be employed when technically feasible and several techniques and devices are currently available. 15 In the case reported, among the different solutions we could not use the Gore Excluder Iliac Branched Endoprosthesis (IBE), because the internal iliac artery was inadequate (instructions for use for IBE requires the internal iliac artery diameter to be between 6.5 and 25 mm) for the device to be deployed. We also rejected application of the parallel graft technique, as in our previous experience the results were poor in terms of primary patency of the chimney grafts. Also, if the patient had accepted open surgery, the internal iliac artery could have been grafted. Therefore, despite some claims that simple coverage without embolization does not increase the risk of secondary interventions, 16 there can still be cases in which HGA perfusion has to be sacrificed to obtain an adequate distal landing zone to prevent Type Ib and Type II endoleaks. As a result, we planned to sacrifice the right HGA to place a straight stent graft landing within the ipsilateral external iliac artery.

In the case reported, there were several elements that constituted risk factors for the development of PSCI after unilateral HGA occlusion (i.e., after right HGA exclusion, the spinal cord blood flow would have been based only or mostly on the left HGA):

1. It is well acknowledged that an extensive longitudinally continuous collateral network exists and accounts for preservation of spinal cord perfusion when intercostal and lumbar segmental arteries (SAs) are interrupted. 17 Recent studies have demonstrated that the total number of intercostal and lumbar SAs sacrificed is a more powerful predictor of the risk of paraplegia than the loss of any specific one. 18 19 In this case, the patency status of the artery of Adamkiewicz was not checked during the first repair. However, given the patient's history (i.e., prior open surgical repair of the infrarenal aorta with occlusion of lumbar and inferior mesenteric arteries; extensive endovascular covering of the descending thoracic aorta with occlusion of multiple intercostal arteries), we believed that the lumbar and intercostal spinal collateral network was almost completely excluded.
2. In the setting of acute complicated Type B dissection, given that the aim of endovascular intervention was primary entry tear closure and that it was mandatory to deploy the stent graft proximally in the nondissected area, we covered the orifice of the LSA. We did not perform any adjunctive revascularization procedures, as the neurologic symptoms at presentation (i.e., paraplegia) resolved and the patient did not develop any complications that could be related to the exclusion of the LSA (i.e., vertebrobasilar insufficiency, stroke, left arm ischemia).
3. The clinical presentation of the acute aortic syndrome with paraplegia led us to hypothesize some aortic collateral branch of great importance within the medullary perfusion pathway. In our view, the postoperative resolution of the neurological symptoms after reopening of anterograde aortic flow could result from the effective supply of collateral perfusion from the pelvic reservoir.

In view of the above considerations, it was mandatory to obtain some form of neurologic monitoring before definitive occlusion of the right internal iliac artery. The recording of evoked potentials to assess spinal cord viability is currently the gold standard for neuromonitoring during open surgery for thoracoabdominal aortic aneurysms. 20 Experience suggests that intraoperative neuromonitoring by means of SSEPs and MEPs is an effective method to detect spinal cord ischemia during descending thoracic and thoracoabdominal aortic surgery. 21 22 A reduction in response amplitude, an increase in latency, a total loss of signal, and the need to increase stimulation voltage are some of the signs that indicate possible ischemia.

SSEPs are obtained by stimulation of the posterior tibial nerve at the medial malleolus, while the waveforms are simultaneously recorded by electrodes placed on the scalp. SSEPs monitoring has been shown to offer an improvement in surgical strategy during thoracoabdominal aortic surgery. However, SSEPs only record the activity of the posterior and lateral columns of the spinal cord, while they fail to detect the function of the anterior columns of the spinal cord. The anterior corticospinal tract is the critical area which, when affected by an ischemic insult, may lead to paraplegia and aortic surgery is more likely to compromise blood flow in the anterior spinal artery (which supplies the motor tracts) than the posterior spinal artery (which supplies the sensory tracts). Thus, SSEPs do not effectively reflect motor function and motor tract blood supply. SSEPs have also been criticized in the past because they can have a slow response to spinal cord ischemia. 23

In view of the above disadvantages, the use of MEPs has proliferated because they tend to have better clinical correlations and lower false negative rates. 24 25 MEPs are elicited either transcranially or by stimulation of the spinal cord directly. Motor responses can then be recorded at three different levels: the spinal cord (spinal MEPs), the nerve (neurogenic MEPs), and the muscle (myogenic MEPs). Indeed, there is evidence that early loss of MEPs identifies patients at higher risk of PSCI who warrant aggressive anesthetic and surgical techniques. 26 Also, current evidence indicates that MEPs strongly predict paraplegia in those patients who totally lose their signals and do not regain them intraoperatively. 27 28

In the presented case, after careful multidisciplinary evaluation, a time of balloon occlusion of 10 minutes was deemed appropriate to rule out the need for HGA revascularization. However, neuromonitoring during extensive endovascular aortic repair requires special considerations since the vascular accesses needed for aortic stent graft placement may result in leg ischemia, thereby eliminating cortical SSEPs and MEPs from the leg. Moreover, anesthetic agents can introduce additional confounding factors as most volatile anesthetic agents depress myogenic responses, and neuromuscular blocking agents can also affect the amplitude of MEPs waves. There is also a lack of randomized controlled trials because it would seem unethical to perform a trial withholding monitoring from patients; for the same reason, it is difficult to determine the true efficacy of SSEPs and MEPs because one would need to avoid corrective actions, which would be difficult to justify ethically. Finally, it should be noted that while false negative results may reflect genuine failure of neuromonitoring techniques potentially leading to devastating consequences, false positive results could trigger unnecessary spinal cord protection interventions which might carry risks themselves. Thus, a careful and comprehensive risk to benefit evaluation to such patients remains crucial.

In conclusion, we herein report a novel application of neuromonitoring to prevent paraplegia after aortoiliac aneurysm endovascular repair.

Currently, there is not any validated method to determine the probability of occurrence of PSCI after HGA sacrifice. Notwithstanding the limited nature of our report, we think that in selected patients, with concomitant presence of strong indications for bilateral HGA preservation and technical difficulties in their preservation, evoked potential recording during iliac endografting represents a useful tool to augment intraoperative management.

## Notes

Editor's Comments We applaud the authors' creative use of neuromonitoring in the nontraditional setting of adjunctive internal iliac artery occlusion. We wonder if the 10-minute occlusion was adequate. We would have preferred a 20-minute test occlusion. We usually identify the artery of Adamkiewicz in advance of major aortic procedures. This can be done effectively by special CT scan techniques.
